# A Case Series of Bowen's Disease Treated with the Combination of Cryosurgery and Ingenol Mebutate and Followed Up with Optical Coherence Tomography

**DOI:** 10.1155/2018/9423949

**Published:** 2018-12-16

**Authors:** Georgios Gaitanis, Theodora Tsironi, Panagiota Spyridonos, Ioannis D. Bassukas

**Affiliations:** ^1^Department of Skin and Venereal Diseases, Faculty of Medicine, School of Health Sciences, University of Ioannina, Ioannina, Greece; ^2^Laboratory of Medical Physics, Faculty of Medicine, School of Health Sciences, University of Ioannina, Ioannina, Greece

## Abstract

Bowen's disease (BD) is a relatively rare* in situ* squamous cell carcinoma (SCC) with a limited potential of becoming invasive. Ingenol mebutate (IM) was relatively successful for the treatment of BD lesions in small case series. Optical coherence tomography (OCT) is a promising method for the diagnosis of cutaneous keratinocytic carcinomas, including BD. Herein we report the treatment of BD with the combination of cryosurgery and IM and the application of OCT imagining in treatment monitoring. Patients treated within a period of 12 months are retrospectively compiled. Treatment consisted of a mild cryosurgery session (liquid N_2_, open spray, and 2 freeze-thaw cycles of 15 sec each) of a field including the BD lesion and a 0.5cm rim and IM application for 4 consecutive days starting at the cryosurgery day. Four patients (3 females; average age: 76.5 years) with 4 lesions (20-70mm maximal diameter; average 36.2mm) were included. Healing was excellent and no relapse was observed at 12 months' follow-up. Baseline OCT revealed a disarranged, thickened epidermis, while a normally layered epidermis overlying a hyperreflective dermis was present after treatment. Conclusively, the combination of cryosurgery followed by IM is a feasible, effective treatment for BD that should be evaluated in further studies.

## 1. Introduction

Bowen's disease (BD) is a relatively rare* in situ* cutaneous squamous cell carcinoma (SCC) with the potential of becoming invasive in a small fraction of cases [1, 2]. BD can acquire extensive dimensions or present in poor healing sites challenging treatment selection [1]. Photodynamic therapy is currently considered the treatment of choice, yet it has been associated with increased relapse rates, the need for repeated treatments [3], and still an appreciable risk of developing an invasive SCC within the treated region [4, 5]. Up to now, ingenol mebutate (IM) has been used in small case series for the treatment of Bowen's disease lesions with relative success [6–11].

Optical coherence tomography (OCT) is a promising imaging modality for the noninvasive evaluation of cutaneous keratinocytic neoplasms, including BD [12]. Preliminary findings in the literature claim the identification of architectural disorganization of the epidermis with a prominent wide spindle layer as the principal OCT diagnostic sign of this latter condition [12].

Herein, we retrospectively report on the feasibility of BD treatment with a 4-day short combination scheme of a cryosurgery session at day 0 followed by 4 daily topical ingenol mebutate applications (days 0-3) and the potential of OCT imaging in treatment monitoring.

## 2. Case Series

All patients included in this study had biopsy-proven BD and gave informed consent according to the principles of the Declaration of Helsinki prior to treatment. This retrospective study was approved by the Institutional Review Board of the University Hospital of Ioannina. The period under evaluation ranged from 1 September 2016 to 31 August 2017 and the files were assessed for last follow-up information at 14 September 2018. BD lesions were imaged prior to biopsy with OCT (NITID, DermaLumics, Madrid, Spain). At least 5 scans of the lesion and of proximal or contralateral healthy skin were acquired. OCT scanning of the treated area was additionally followed up at 3, 6, and 12 months after treatment. All authors evaluated the scans independently in the monitor of the OCT device in a darkened room.

A four-day treatment protocol was administered combining one cryosurgery session and 4 daily ingenol mebuate (Picato® Gel, 500MCG/G) applications. Treatment started with a relatively mild cryosurgery session (liquid N_2_, open spray, 2 freeze-thaw cycles of 15 sec each) of a skin area including the BD lesion and a 0.5cm rim around it (day 0 of treatment). For larger BD lesions, freezing was performed in slightly overlying 2x2cm skin sections. The patient was instructed to apply ingenol mebutate once daily on the cryosurgery pretreated area starting already at the evening of the same day 0 of the treatment and for the 3 following days: 1-3 (4 once daily applications in 4 consecutive days in total). The patients were evaluated 1 week after the cryosurgery session and at 1, 3, 6, and 12 months after treatment. Primary treatment target was clearance of the lesions (clinical complete response); residual disease <25% of the initial lesion was considered as partial response.

**Figure 1:**
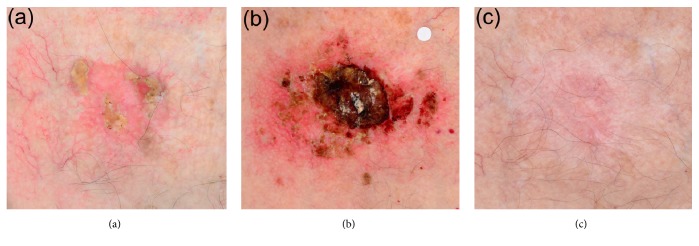
A 33 mm Bowen's disease lesion of the sternum (Patient 4). Cross-polarized photography is applied to enhance the visualization of the vascular pattern. Panel (a): the lesion at baseline. Panel (b): the area of the lesion at day 7 of treatment (one week after treatment onset with cryosurgery at day 0 and 4 daily applications of ingenol mebutate in days 0-3 of treatment). The white label is included for calibration purposes. Panel (c): the site of the lesion at 12 months follow-up. A slightly hypopigmented shallow scar is evident with excellent skin healing (hair follicle preservation) in a skin location of overall problematic healing features (sternum).

A total of 4 patients (3 females and 1 male; average age: 76.5 years) with 4 BD lesions (20-70mm maximal diameter; average: 36.2mm) were treated with cryosurgery followed by ingenol mebutate according to the above combination scheme (Table 1 and Figure 1). All patients experienced vivid local inflammation with redness, blistering, and scabbing which intensified during treatment with ingenol mebuate. However, the local tissue reaction was reported to peak at day 7 of treatment (Figure 1(b)) and subsequently started to subside. At 1-month follow-up, some redness was evident at the site of treatment, with concomitant hypopigmentation. Scaring was minimal and gradually improved during further follow-up. Taking treatment outcomes together, clearance (complete response) was achieved in all 4 BD cases and no relapses were observed at 12 months' follow-up (Figure 1(c)).

**Figure 2:**
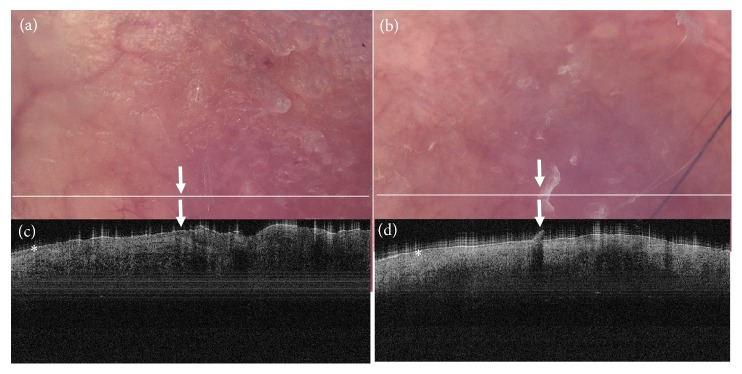
Dermoscopic images of the lesion acquired with the digital dermatoscope incorporated to the NITID device and the corresponding OCT image of Bowen's lesion shown in Figure 1. The white line on the dermatoscopic image corresponds to the section scanned by OCT. Panels (a, b): the lesion at baseline. The left half of the OCT scan includes a healthy skin segment (control) featuring a well-defined epidermis (asterisk). At the transition of healthy skin to the lesion, the typical for Bowen's disease thickening and disarrangement of the epidermis becomes evident (right to the arrow). Panels (b, d): the site of the lesions at the 12^th^ month follow-up. There is normalization of the epidermis in the dermoscopic and the OCT images (asterisk). The focal hyperkeratosis evident in the dermatoscopic image (Panel (b), arrow) is also evident in the OCT image as a disruption in the epidermis (Panel (d), arrow) which, however, causes no concern as the epidermis is homogenous throughout the scanned section.

In accordance with findings by other authors [12], however with different OCT devices, OCT imaging of the BD lesions at baseline revealed a thickened epidermis without a prominent granular layer in all cases (Figure 2(c)). Early after treatment, a normally layered hyporeflective epidermis was present overlying a hyperreflective dermis. Residual, clinically not evident edema could probably account for this finding. At 12 months' follow-up, the posttreatment hyperreflectiveness of the dermis had subsided, leaving behind a pattern almost indistinguishable from normal skin (Figure 2(d)).

## 3. Discussion

We presently report the feasible and efficacious treatment of relatively large BD lesions (20 – 70mm in maximal diameter) with 4-day cryosurgery-ingenol mebutate combination consisting of an initial cryosurgery session followed by 4 daily ingenol mebutate applications. In addition, the observations of OCT monitoring of the treated sites are presented. Cryosurgery monotherapy is considered a suitable treatment modality for selected BD lesions [1]. Its mode of action is not restricted to the direct destruction of the malignant tissues, but it also addresses the induction of a proapoptotic state in malignant cells, the destruction of tumor vasculature, and antitumoral immune stimulation [13]. We suggest that the pathophysiological effects of the cryoablation may synergize with the cellular and tissue sequels of the concomitant ingenol mebutate application on the skin [14, 15] which might predict an enhanced therapeutic efficacy of the above combination. Also, physical disruption of the epidermal barrier with cryosurgery definitely increases ingenol mebutate penetration into the skin and probably enhances efficacy. We have previously evaluated in our department alternative combination therapeutic modalities for BD with overall satisfactory results, however, of substantially protracted application time in comparison to the present proposal. In an overview of BD treatment with immunocryosurgery, that is, the 5 weeks combination of daily imiquimod and a session of relatively mild cryosurgery at day 14 of the cycle [16, 17], sustained clearance was achieved in >95% of 35 BD lesions in 29 patients, including some particularly large BD sites (>100mm in maximal diameter) or lesions in poor healing sites like the shins. However, with the accumulation of extensive experience in the treatment of BCC [18], we acknowledge that lengthy topical treatment protocols (e.g., 35 days of a typical immunocryosurgery cycle) may limit the compliance particularly of the usually older patients with BD. Therefore, highly effective minimally invasive treatment modalities of shortened application periods are anticipated to further improve overall effectiveness of nonsurgical modalities for skin malignancies. In published cases of ingenol mebutate-treated Bowen's diseases, there is an effort to increase efficacy through the combination with prior laser ablation [10] or ingenol mebutate application under occlusion even in transplanted patients [9, 11] without entailing safety issues in this special setting. Our present findings confirm the feasibility of the proposed topical combination modality for BD, as it seems to combine promising efficacy and acceptable side effects profile. It is worth noting that a similar to the present combination modality of cryosurgery and IM has been evaluated in the treatment of actinic keratosis on the dorsal hands and was compared to cryosurgery monotherapy. The authors confirmed increased efficacy in addition to the feasibility of the proposed approach [19].

The OCT features of the treated area include a well-defined homogenous epidermis band over a normal dermis, findings that constitute an unequivocal background for the monitoring for BD relapses after efficient tissue sparring treatment modalities.

In conclusion, the present case series demonstrates the feasibility of a 4-day combination modality of cryosurgery and ingenol mebutate for BD as well as the potential of OCT imaging to improve the noninvasive monitor of treatment outcomes. Both goals should be evaluated in further studies.

## Figures and Tables

**Table 1:** Patients, tumors, and treatment characteristics. Patients 1 and 3 required half a tube per application of the commercially available ingenol mebutate gel (Picato® Gel, 500MCG/G), while patients 2 and 4 applied the whole tube per application.

Patient/Tumor Number	Gender	Age	Co-morbidities	Concomitant medication	Tumor Localization	Maximal diameter (mm)
1	F	79	Pacemaker, arterial hypertension, diabetes mellitus, hypothyroidism	Aminosalycilic acid, propranol, amlodipine, ramipril, atorvastin, dapagliflozin, vildagliptin, glycazide, thiamazole	Nose	20
2	F	81	Asthma, atrial fibrilation	Flixotide, budesonide, formoterol, aminosalycilic acid, isoprenaline	Preauricular	70
3	F	68	Diabetes	Sitagliptin	Mandible	22
4	M	78	Atrial fibrilation, arterial hypertension,	Carvedilol, propafenone, rivaroxaban, olmesartan	Chest	33
